# Supplementary material for: Dispensing of Non-Prescribed Antibiotics from Community Pharmacies of Pakistan: A Cross-Sectional Survey of Pharmacy Staff’s Opinion
Source: Antibiotics (Basel). 2021 Apr 22;10(5):482. doi: 10.3390/antibiotics10050482 (PMC8143445; doi:10.3390/antibiotics10050482)
Supplement: Supplementary file 1 [file antibiotics-10-00482-s001.pdf]

Questionnaire

Part 1: Demographics of respondents

1. Age________________________ years 2. Gender___________________________

3. Educational status_________________ 4. Experience________________________

5. Status in Pharmacy : Employee ☐ Manager ☐ Proprietor ☐

6. Prescriptions dispensed ___________________________ /day

7. Total antibiotics dispensed (number of customers) ___________________/day

8. Dispensed no prescribed antibiotics (number of customers) __________________/day

Part 2: Knowledge and Attitude

Please tick (✓) the relevant

1. Community pharmacies in Pakistan are authorized for DoNA

☐ Yes ☐ No ☐ Don't Know

2. DoNA is common in community pharmacies of Pakistan

☐ Yes ☐ No ☐ Don't Know

3. Dispensing of prescribed only medicine without prescription causes health issues

☐ Yes ☐ No ☐ Don't Know

4. DoNA contributes to antimicrobial resistance (AMR) development

☐ Yes ☐ No ☐ Don't Know

5. AMR has become a challenging issue of public health

☐ Yes ☐ No ☐ Don't Know

6. DoNA is a cause of irrational use of antibiotics by patients

☐ Yes ☐ No ☐ Don't Know

7. Pharmacy retailers should stop DoNA

☐ Yes ☐ No ☐ Don't Know

8. I recommend doctor's consultation to the patients before use of any antibiotic

☐ Yes ☐ No

9. On my refusal to DoNA, patient will try to get from another pharmacy

☐ Yes ☐ No ☐ Don't Know

10. DoNA reduces the economic burden of patients

☐ Yes ☐ No ☐ Don't Know

Part 3 : Practices of DoNA

11. Reasons for DoNA (Please includes all possible answers or mention other)

☐ Pharmacy retailers have proper knowledge about use of antibiotics

☐ Patients visit physicians only in the case of serious infection
☐ Increases the profit and sales
☐ Lack of patients' affordability for doctors' consultation fee
☐ Fear of customer loss
☐ Unawareness of pharmacy staff toward policy and regulations to halt DoNA
☐ Other____(Please mention)

12. Please mention the name of commonly dispensed non prescribed antibiotic

1. ____________________________ 2. ____________________________

3. ____________________________ 4. ____________________________

5. ____________________________ 6. ____________________________

7. ____________________________ 8. ____________________________

9. ____________________________ 10. ____________________________

11. ____________________________ 12. ____________________________

13. ____________________________ 14. ____________________________

13. Please mention the dosage of commonly dispensed non prescribed antibiotic.

1. ____________________________ 2. ____________________________

3. ____________________________ 4. ____________________________

5. ____________________________ 6. ____________________________

14. Please mention the diseases for which non prescribed antibiotic are dispensed.

1. ____________________________ 2. ____________________________

3. ____________________________ 4. ____________________________

5. ____________________________ 6. ____________________________

7. ____________________________ 8. ____________________________

9. ____________________________ 10. ____________________________

Please tick (✓) the relevant

Measures	Response
15. Before DoNA, I inquire the drug allergies to patients	Always ☐ Very often ☐ Some time ☐<br>Rarely ☐ Very rarely ☐ Never ☐

16. Before DoNA , I inquire about the renal condition of patients	Always ☐ Very often ☐ Some time ☐<br>Rarely ☐ Very rarely ☐ Never ☐
17.Before DoNA, I ask the patient about other disease/s or therapies	Always ☐ Very often ☐ Some time ☐<br>Rarely ☐ Very rarely ☐ Never ☐
18. At the time of DoNA, I inform the patients about possible side effects	Always ☐ Very often ☐ Some time ☐<br>Rarely ☐ Very rarely ☐ Never ☐
19. At the time of DoNA, I counsel the patients for medication adherence and compliance	Always ☐ Very often ☐ Some time ☐<br>Rarely ☐ Very rarely ☐ Never ☐
20.I don't dispense non-prescribed antibiotics for children	Always ☐ Very often ☐ Some time ☐<br>Rarely ☐ Very rarely ☐ Never ☐
21.I don't dispense non-prescribed antibiotics for pregnant women	Always ☐ Very often ☐ Some time ☐<br>Rarely ☐ Very rarely ☐ Never ☐

Part 4 : Suggestions to halt DoNA

Please tick (✓) the relevant

22. What you suggest to control DoNA from community pharmacies

☐ Pharmacy retailers should penalized for DoNA

☐ Vigilance of pharmacies by drug inspectors should be more strong

☐ Pharmacy retailers need continuous training about AMR

☐ Other____(Please mention)

23. What should be the role of pharmacy retailers to halt the use of non-prescribed antibiotics

☐ Educate patients about the outcome and AMR due DoNA

☐ Educate and train the staff of pharmacy about the outcome and AMR due DoNA

☐ Arranging awareness seminars during the meetings of Chemist and Druggist Associations

☐ Arrange public awareness campaigns

☐ Other____(Please mention)

Thank you so much for your precious time.
